# Prognostic significance of functional somatic symptoms in adolescence: a 15-year community-based follow-up study of adolescents with depression compared with healthy peers

**DOI:** 10.1186/1471-244X-12-90

**Published:** 2012-07-27

**Authors:** Hannes Bohman, Ulf Jonsson, Aivar Päären, Lars von Knorring, Gunilla Olsson, Anne-Liis von Knorring

**Affiliations:** 1 Department of Neuroscience, Child and Adolescent Psychiatry, Uppsala University, SE-75185, Uppsala, Sweden; 2 Department of Neuroscience, Psychiatry, Uppsala University, Uppsala, Sweden

**Keywords:** Adolescent depression, Long-term follow-up, Functional somatic symptoms, Anxiety and suicidal behavior

## Abstract

**Background:**

There is a lack of population-based long-term longitudinal research on mental health status and functional physical/somatic symptoms. Little is known about the long-term mental health outcomes associated with somatic symptoms or the temporal relationship between depression and such symptoms. This 15-year study followed up adolescents with depression and matched controls, screened from a population-based sample, who reported different numbers of somatic symptoms.

**Methods:**

The total population of 16–17-year-olds in Uppsala, Sweden, was screened for depression in 1991–1993. Adolescents who screened positive and an equal number of healthy controls took part in a semi-structured diagnostic interview. In addition, 21 different self-rated somatic symptoms were assessed. Sixty-four percent of those adolescents participated in a follow-up structured interview 15 years later.

**Results:**

Somatic symptoms in adolescence predicted depression and other adult mental disorders regardless of the presence of adolescent depression. In adolescents with depression, the number of functional somatic symptoms predicted, in a dose response relationship, suicidal behavior, bipolar episodes, and psychotic episodes as well as chronic and recurrent depression. Contrary to expectations, the somatic symptoms of abdominal pain and perspiration without exertion better predicted depression than all DSM-IV depressive symptoms. Abdominal pain persisted as an independent strong predictor of depression and anxiety, even after controlling for other important confounders.

**Conclusions:**

Somatic symptoms in adolescence can predict severe adult mental health disorders. The number of somatic symptoms concurrent with adolescent depression is, in a stepwise manner, linked to suicidal attempts, bipolar disorders, psychotic disorders, and recurrent and chronic depression. These findings can be useful in developing treatment guidelines for patients with somatic symptoms.

## Background

Functional somatic symptoms (i.e., physical symptoms without a known medical explanation) are common in the health care system. Approximately one third of patients in primary health care suffer from multiple somatic symptoms, representing the main reason for consultation [1,2]. These patients are distinguishable from patients with explained medical disorders. They have a higher level of health care utilization and more prolonged sick leave [3]. They are more likely to suffer from a difficult patient-doctor relationship, are less satisfied with treatment, and more often feel they are not being taken seriously by the doctor [4,5].

Functional somatic symptoms are linked to depression, anxiety disorders, and social stress [1]. If depressed patients seek help for physical symptoms, their depression diagnosis is often delayed as is treatment [6]. Furthermore, concurrent somatic symptoms can be a marker for a depressive disorder with higher severity and worse prognosis [7,8]. Depressed patients with functional somatic symptoms respond less often to medical treatment [9,10], and they reach remission less frequently [11]. In older adults, depression with somatic symptoms is linked to increased mortality. In patients with depression and cardiovascular disease, somatic and not affective/cognitive symptoms of depression are predictive of earlier death or cardiac events [12].

In an earlier study that was the basis for this study; functional somatic symptoms and depression were investigated in adolescents [13]. Somatic symptoms were much more common among adolescents with depression. For each additional symptom in those adolescents, severity of depression and occurrence of severe concurrent mental disorders increased. Among the quarter of adolescents that had more than five somatic symptoms, current suicidal behavior increased nine-fold compared to depressed adolescents without somatic symptoms. The same relationship was observed for somatic symptoms and drug abuse, disruptive behavior, and multiple stressful relationships. Functional somatic symptoms in depression marked a subgroup with increased severity. These findings raise questions about long-term outcomes in adolescents with functional somatic symptoms.

Previous studies have shown an association between functional somatic symptoms and adverse mental health outcomes. However, the number of studies is small. Little is known about the prospective mental health of adolescents with co-occurring somatic symptoms and depression [9]. The lack of population-based long-term longitudinal studies has been stressed in previous reviews, and there is conflicting evidence as to whether depression is a risk factor for or a consequence of functional somatic symptoms [14].

### Aim

We aimed to investigate adult mental health outcomes of adolescents with functional somatic symptoms. A second aim was to examine the causal relationship between somatic symptoms and depression. A third aim was to explore whether particular somatic symptoms are predictive of adverse mental health in adulthood. We hypothesized that the number of somatic symptoms in adolescents with depression would be predictive of mental health outcomes in adulthood.

## Methods

### Study population and procedure

In 1991–1993, all first-year students (16–17 years old) in upper secondary school in Uppsala, Sweden, a university town of 200,000 inhabitants, were asked to participate [15] in a depression screening. School dropouts were also invited. Of the 2,465 adolescents, 93% (n = 2,300) participated in the screening that included two self-evaluations of depression: the Beck Depression Inventory-Child and the Centre for Epidemiological Studies-Depression Scale for Children [16,17]. Students with high scores (BDI-C ≥ 16, CES-DC ≥ 30) or who reported a suicide attempt were interviewed with the revised adolescent version of the Diagnostic Interview for Children and Adolescents (DICA-R-A) according to the Diagnostic and Statistical Manual of Mental Disorders (DSM-III-R) criteria [18].

**Figure 1:**
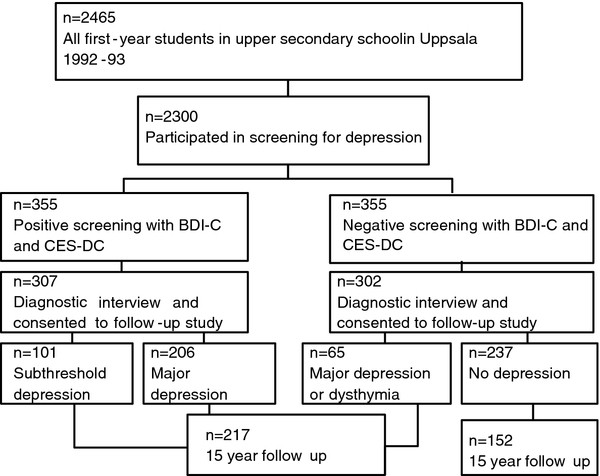
**Outline of the procedure at adolescence and at follow-up in adulthood.** *The Beck Depression Inventory - Child (BDI-C) and the Center for Epidemiological Studies – Depression Scale for Children (CES-DC) were used. Positive screening was defined as BDI-C ≥16, or CES-DC ≥30 and BDI-C ≥11, or attempted suicide.

Three hundred fifty-five students were selected for the interview. Same-sex peers of the same age and in the same school class who had low screening scores were selected for a comparison group. A total of 609 adolescents were interviewed and completed the Somatic Symptom Checklist Instrument (SCI). About 15 years later, the participants who had consented to a follow-up study were contacted and asked for a follow-up interview. Those who met the criteria for manic or hypomanic episodes according to the DICA-R-A (n = 40) were excluded from the present study, leaving a total of 569 participants. Of these, 64.8% (n = 369) took part in the follow-up interview; 3.6% (n = 21) had not given their consent to be contacted for a follow-up, 5.8% (n = 36) had emigrated or lived abroad, 0.5% (n = 3) were not alive, 6.8% (n = 38) could not be reached, and 18.4% (n = 107) either refused to participate or agreed to participate but could not find the time. At follow-up, participants ranged in age from 30–33 years (M = 31.6, SD = 0.8). The procedure is outlined in Figure 1.

### Definition of depression groups

The depression group included those with adolescent major depressive disorder (MDD) and subclinical depression (dysthymia or positive at screening but no depressive disorder determined in the interview). Long-term depression in adolescence was defined as a major depression most of the time lasting a minimum of one year, or a major depressive episode preceded or followed by dysthymia (double depression) for a minimum of one year. Chronic depression in adulthood was defined as a major depressive episode lasting more than 2 years. For this particular study, participants were divided into five groups based on their adolescent status: one group of controls without depression (n = 152) and four groups with different numbers of somatic symptoms (n = 217). Among the adolescents with depression, 16% (n = 35) had no somatic symptoms, 34% (n = 79) had 1–2 somatic symptoms, 27% (n = 59) had 3–4 somatic symptoms, and 23% (n = 50) had 5 or more somatic symptoms. The participation rate at follow-up did not differ significantly between the groups.

### The adolescents without depression

Among the adolescents without depression (n = 152), 47% had no somatic symptoms, 39% had 1–2, 11% had 3–4, and 3% had 5 or more somatic symptoms. The adolescents without depression rarely suffered from co-morbid mental disorders according to DSM-III criteria compared to the adolescents with depression [19]. The adolescents without depression were healthier than the general population.

### Baseline evaluation

The SCI assesses 22 items of various physical symptoms (Table 1)*.* The complaints are graded according to frequency (0 = never, 1 = monthly, 2 = weekly, 3 = several times a week, and 4 = daily) and intensity (0 = no problems, 1 = minor, 2 = moderate, 3 = troublesome, and 4 = extremely troublesome). A somatic symptom was recorded when its multiplied frequency and intensity score was ≥6 (e.g., 2 × 3: weekly and troublesome symptoms). This level excluded less severe cases and the possibility that monthly premenstrual symptoms were recorded as positive. The questionnaire has been used in previous studies [20]. Allergies were not associated with depression at baseline and were excluded in the calculations.

**Table 1:** Somatic symptoms at baseline and follow-up with odds ratios for somatic and DSM-IV depressive symptoms

**Symptom Check List**	**D n = 217 (%)**	**C n = 152 (%)**	**p**	**OR**	**CI**
Headache	34.1	11.9	<0.001	3.8	2.2-6.7
Feeling chilly	27.6	13.2	<0.001	2.5	1.4-4.4
Eye tiredness	21.7	7.3	<0.001	3.5	1.8-7.0
Abdominal pain	16.6	3.3	<0.001	5.8	2.2-15.2
Dizziness	13.8	2.0	<0.001	7.9	2.4-26.4
Nausea	12.0	2.6	<0.001	5.0	1.7-14.7
Perspiration	11.1	4.6	<0.05	2.5	1.1-6.1
Breathing problem	7.4	2.0	<0.05	3.9	1.1-13.7
Polyuria	6.9	0.7	<0.01	11.1	1.5-85.2
Limb pain	6.5	3.3	n.s.	2.0	0.7-5.7
Itching	5.5	1.3	<0.05	4.4	1.0-19.8
Dry mouth	5.1	2.6	n.s.	2.0	0.6-6.3
Tiredness	54.8	22.5	<0.001	4.2	2.6-6.6
Insomnia	29.0	5.3	<0.001	7.3	3.4-15.8
Appetite problem	12.0	2.6	<0.001	5.0	1.7-14.6
DSM-IV Symptoms of Major Depression					
Tiredness	64.3	21.3	<0.001	6.7	4.1-10.7
Insomnia	40.5	9.0	<0.001	6.9	3.7-12.6
Appetite problem	62.1	32.2	<0.001	3.5	2.3-5.5
Dysphoria	83.3	34.2	<0.001	9.6	5.9-15.5
Anhedonia	58.1	9.7	<0.001	13.0	7.2-23.5
Psychomotor retardation/agitation	63.9	23.9	<0.001	5.6	3.6-8.9
Troubled thoughts	66.6	33.5	<0.001	6.6	4.1-10.4
Suicidal thoughts	27.3	1.9	<0.001	19.0	5.8-61.9
Worthlessness	67.0	10.3	<0.001	17.6	9.8-31.6

D = Depression group (adolescents with depression), C = Controls (non-depressed), OR = Odds Ratio, CI = Confidence Interval.

Information on child and adolescent mental disorders was taken from the DICA-R-A. Adolescents meeting the criteria for a lifetime diagnosis of conduct/oppositional defiant disorder were categorized as having disruptive disorder. Adolescents with a childhood diagnosis of separation anxiety, overanxious disorder, or avoidant disorder were categorized as having childhood anxiety disorder.

The original study included the Children’s Life Event Inventory [21]. Items about parental unemployment as well as serious conflict with and between parents were selected. Details of the baseline characteristics have been published previously [19].

### Follow-up evaluation

Participants were interviewed with the Mini International Neuropsychiatric Interview Plus at follow-up [22]. Some disorders (e.g., MDD) were assessed any time from age 19 to follow-up, while current diagnoses were used for other disorders (e.g., anxiety disorders). See Table 2 for details. To further enhance the participants’ recall of depression during the investigated period, a life-chart procedure with additional questions about education, occupation, life events, depression, and treatments was used. The participants were asked about suicidal ideation and suicide attempts during the period from age 19 to follow-up. Details of the procedure have been published previously [23].

**Table 2:** Adult mental health outcomes in adolescents with depression and somatic symptoms compared with non-depressed controls

	**Non- depressed n = 152 %**	**Adolescents with depression n = 217**				**Linear by linear correlation in depression subgroups**
		**0 somatic symptoms n = 35%**	**1-2 somatic symptoms n = 73%**	**3-4 somatic symptoms n = 59%**	**≥5 somatic symptoms n = 50%**	
Major depressive episode^a^	31.1	42.9	54.8**	62.7***	76.0***	p < 0.01
Recurrent depression or long episode (≥2 episodes/>6 months)^a^	21.9	34.3	42.5**	50.8***	68.0***	p < 0.001
Any chronic depression^a^	3.3	11.4*	11.0*	13.6**	30.0***	p < 0.05
Manic/hypo manic episode^a^	2.0	5.7	9.6*	6.8	22.0***	p < 0.05
**Any mood disorder**^a^	**34.4**	**44.1**	57.5**	66.1***	80.0***	**p < 0.001**
Panic disorder, agoraphobia^a^	11.9	20.0	20.5	27.1**	44.0***	p < 0.05
Specific phobia^a^	17.9	25.7	23.3	23.7	28.0	n.s.
Social phobia^**b**^	7.3	8.6	23.3**	11.9	22.0**	n.s.
PTSD^**b**^	0	2.9 *	2,7*	0.0	8.0***	n.s.
GAD^**b**^	4.6	5.7	11.0	13.6	18.0**	n.s
OCD^**b**^	1.3	2.9	5.5	3.4	14.0***	n.s.
**Any anxiety disorder**^a,b^	**22.5**	**34.3**	41.1**	42.4**	58.0***	**p < 0.05**
Drug abuse^a^	1.2	0	2.9	3.7	6.1*	n.s.
Alcohol abuse^a^	3.0	16.1**	4.3	9.3	4.1	n.s.
Any psychosis^a^	1,3	0,0	2.7	3.4	8.0*	n.s.
Any somatoform disorder^b^	2.6	5.7	6.8	8.5*	26.0***	p < 0.01
**Any mental disorder**^a,b^	**45.0**	**60.0**	65.8**	74.6***	86.0***	**p < 0.01**

*Notes*: ^a^ = Any time from age 19 to follow-up. ^b^ = Current at follow-up.

* = *p* < 0.05; **  *= p* < 0.01; *** =  *p* < 0.001 (Referring to comparisons with the non depression group, χ^2^ calculation.

### Data analyses

The chi-square test was used to contrast the groups of adolescents with depression with the non-depressed group. Linear by linear association was used to compare the depressed adolescents with different numbers of somatic symptoms to mental health outcomes. Conditional logistic regression analysis adjusted for sex was used to investigate if particular somatic symptoms predicted adverse mental health. The 15 most common somatic symptoms from the SCI were included. Two different dependent variables were selected: more than two recurrent episodes or a recurrent episode of depression lasting more than six months, and any anxiety disorder at follow-up. Successive elimination of non-significant factors resulted in three depression and two anxiety variables with significant odds ratios.

Previous studies have reported that somatic symptoms predict adverse future health better than affective and cognitive symptoms [12]. To compare the predictive value of somatic symptoms versus affective and cognitive symptoms, a second model was created.

In a previous study, we investigated the predictors of long-term mental health outcomes using logistic regression analysis [23]. The present study included a third model in which long-term depression (double depression vs. episodic MDD and subclinical depression) together with somatic symptoms was added. This model also included family adversities (conflict between parents, conflict with parents, physical abuse at home, economic hardship, and parental unemployment), suicidal behavior, disruptive disorder, drug abuse, and multiple stressful relationships. These items were strongly associated with adolescent depression and increased number of somatic symptoms at baseline [13].

To investigate the relation between adolescent long-term depression, multiple somatic symptoms, and adult mental health outcomes, univariate analyses were performed. Depressed adolescents were divided into two groups: those with ≥5 and those with <5 somatic symptoms. Long-term versus no long-term depression was analyzed for both groups. Any mood and any non-mood disorders were studied as dependent variables.

*P*-values below 0.05 in two-tailed tests were considered significant for all statistical analyses. SPSS 19.0 for Macintosh was used.

### Ethics

After a complete description of the study was provided for participants, written informed consent was obtained. The local ethical vetting board of Uppsala University, Sweden, approved the study.

## Results

At baseline, dizziness, polyuria, insomnia, tiredness, and abdominal pain were most strongly associated with depression (Table 1). There was a linear relationship between number of somatic symptoms in adolescence with depression and the risk of mental disorders as adults for any disorder (p < 0.001), any mood disorder (p < 0.001), recurrent depression (p < 0.001), chronic depression (p < 0.05), bipolar disorders (p < 0.05), any anxiety disorder (p < 0.05), panic disorder (p < 0.05), and somatoform disorders (p < 0.01). Adolescents with depression and ≥ 5 somatic symptoms (23% of the depressed) had marked adverse adult mental health outcomes compared to the other groups (Table 2).

There was a strong overrepresentation of adult suicidal behavior in adolescents with depression who suffered ≥5 functional somatic symptoms, compared to the controls (p < 0.001). However, there was also an overrepresentation of adolescents without somatic symptoms (p < 0.05) (Table 3). There was a linear relationship between suicidal behavior in adolescents with somatic symptoms (1 to ≥ 5) including suicidal thoughts (p < 0.05), plans (p < 0.01), and attempts (p < 0.05) (results not shown).

**Table 3:** Adult suicidal behavior in adolescents with depression and somatic symptoms compared with non-depressed controls

	**Non depressed n = 152 %**	**Depressed adolescents with different number of somatic symptoms n = 217**				**Linear by linear correlation in depression subgroups**
		**0**	**1-2**	**3-4**	**≥5**	
		**n = 35%**	**n = 73%**	**n = 59%**	**n = 50%**	
Suicidal ideation in adulthood						
Thoughts of being better dead^a^	14.6	34.3**	26.6*	33.9**	48.0***	n.s.
Suicidal thoughts^a^	9.0	20.6*	12.3	16.9	28.0***	n.s.
Active suicide plans^a^	4.8	11.8	5.5	6.8	22.0***	n.s.
Suicide attempt^a^	2.6	11.8*	4.1	5.1	16.0**	n.s.
Hospitalized for suicide attempt^a^	1.3	5.9	1.4	3.4	8.0*	n.s.

*Notes*: ^a^ = Any time from age 19 to follow-up.

* = *p* < 0.05; ** =  *p* < 0.01; *** =  *p* < 0.001 (Referring to comparisons with the non-depression group, χ^2^ calculation).

The controls with somatic symptoms in adolescence were more likely than controls without somatic symptoms to report any disorder, any mood disorder, MDD, any anxiety disorder, and self-harm behavior (p < 0.05) (Table 4).

**Table 4:** Adult mental health outcomes in non-depressed adolescents with and without somatic symptoms

**Adult diagnosis**	**Non depressed without somatic symptoms n = 71**	**Non depressed with somatic symptoms n = 81**		
	**%**	**%**	**OR**	**CI**
Major depressive episode^a^	22.5	38.3*	2.2	1.06–4.45
Recurrent depression or long episode (≥2 episodes or longer than 6 months)	9.9	21.0	2.5	0.96-6.36
Any chronic depression^a^	0.0	6.2*	-	-
Manic episode/ hypomanic^a^	1.4	2.5	1.8	0.16-20.22
**Any mood disorder**^a^	**25.4**	42.5*	**2.2**	**1.09–4.36**
Panic disorder, agoraphobia^a^	5.6	17.5*	3.6	1.11–11.36
Specific phobia^a^	14.1	21.1	1.6	0.71-3.88
Social phobia^b^	5.6	8.8	1.6	0.45-5.73
PTSD^b^	0.0	0.0	-	-
GAD^b^	2.8	6.2	2.3	0.43-12.24
OCD^b^	0.0	2.5	-	-
**Any anxiety disorder**^a b^	**14.1**	30.0*	**2.6**	**1.15–5.95**
Psychosis^a^	0.0	2.5	-	-
Somatoform disorders^b^	4.2	1.2	0.3	0.29-2.82
**Any non mood disorder**^a^	22.5	35.0	1.9	0.90-3.81
**Any mental disorder**^a^	**32.2**	53.8*	**2.2**	**1.11–4.12**
Self harm^a^	0	7.4*	-	-
Suicidal thoughts^a^	4.2	12.5	3.23	0.85-12.28
Suicidal plans^a^	1.4	6.3	4.67	0.53-40.94
Suicidal attempts^a^	1.4	3.8	2.73	0.28-26.83
Hospitalization due to suicidal attempts^a^	0.0	2.5	-	-

*Notes*: ^a^ = Any time from age 19 to follow-up. ^b^ = Current at follow-up.

*p < 0.05; (Referring to comparisons with the non-somatic symptom group).

Baseline characteristics were investigated for the controls with and without somatic symptoms. Family adversities (conflict between parents, conflict with parents, physical abuse at home, economic hardship, and parental unemployment) and mental/physical health problems (suicidal ideation, physical illness, childhood anxiety, and disruptive disorder) were included. Problems were uncommon in both groups, but two variables differed. Adolescent controls with somatic symptoms compared to controls without symptoms had more often experienced conflict between parents (1.4% vs. 16.3%, p < 0.01) and childhood anxiety (5.6% vs. 25%, p < 0.01). Because childhood anxiety might explain the presence of adult mood disorders in the non-depressed, analyses were performed to investigate this. The presence of at least one somatic symptom during adolescence predicted any mood disorder at follow-up (OR 2.1, CI 1.04–4.37), even when the presence of any childhood anxiety disorder was included as a covariate. However, any childhood anxiety disorder did not predict any mood disorder (OR 1.2, CI 0.46–3.03) when the presence of at least one somatic symptom was included as a covariate.

Abdominal pain, perspiration without exertion, and feeling chilly predicted recurrent or extended episodes of depression in adulthood. Abdominal pain remained a significant predictor together with long-term depression when controlling for DSM-IV criteria for MDD, family adversities, and adolescent behavior problems (suicidal behavior, disruptive behavior, and drug abuse). Abdominal pain also predicted anxiety disorders when controlling for DSM-IV criteria for MDD, family adversities, and adolescent behavior problems (Table 5).

**Table 5:** Prediction of depression and anxiety in adulthood for adolescents with depression and somatic symptoms

**Outcome: ≥2 episodes or >6 month duration of MDD in adulthood**	**Model 1**		**Model 2**		**Model 3**	
	**OR**	**95% CI**	**OR**	**95% CI**	**OR**	**95% CI**
Abdominal pain	3.2	1.41–7.42	3.3	1.40–7.56	3.2	1.35–7.27
Perspiration	3.1	1.12–8.35	3.4	1.2–9.45		
Feeling chilly	1.9	1.00–3.49				
Anhedonia			2.5	1.43–4.51		
Long-term depression (>1 year duration)					3.5	1.91–6.28
Outcome: any anxiety disorder at follow-up	Model 1		Model 2		Model 3	
	OR	CI	OR	CI	OR	CI
Abdominal pain	2.6	1.19–5.57	3.0	1.33–6.77	2.3	1.02–5.01
Tiredness	2.0	1.13–3.52	2.4	1.29–4.34		
Headache			0.4	0.27-0.96		
Worthlessness			2.1	1.09–3.90		
Troubled thoughts			1.9	1.01–3.59		
Long-term depression (>1 year duration)					3.9	2.12–7.04

Notes: Only significant results are shown.

Model 1 includes the 15 most common somatic symptoms according to SCI and sex.

Model 2 adds 9 DSM-IV depression criteria.

Model 3 adds long-term depression (double depression vs. dysthymia, episodic major depression, and subclinical depression), family adversities (conflicts between parents, conflicts with parents, physical abuse at home, economic hardship and parental unemployment), suicidal behavior (suicidal plans or attempts), disruptive disorder (conduct disorder, oppositional defiant disorder) and drug abuse.

Long-term depression was common in the group with ≥5 somatic symptoms (60%). Within this group, long-term depression did not significantly predict mood disorders at follow-up (84.4% vs. 72.2%, p = 0.30). In the group with <5 somatic symptoms, long-term depression strongly predicted any mood disorder in adulthood (83.0% vs. 50.0%, p < 0.001). Long-term depression did not provide a significantly better prediction of any non-mood disorder in the subgroup with ≥5 somatic symptoms (71.9% vs. 55.6%, p = 0.24).

## Discussion

This 15-year follow-up study of adolescents with depression and healthy controls, which were screened from a Swedish population, demonstrates a strong relationship between the presence of somatic symptoms in adolescence and adverse mental health outcomes in adulthood. The relationship was most pronounced when somatic symptoms were concurrent with depression, but the relationship also existed in the controls that had no experience of previous depression. The result that somatic symptoms predicted adverse health outcomes is in line with other prospective studies [24-27]*.* Most previous studies have focused on depressive and anxiety disorders as outcome measures, often in relation to treatment and over a shorter time period.

In our study, using structured interviews, the relationship between mental health and somatic symptoms was evident also for more severe mental disorders (e.g., psychotic and bipolar disorders as well as suicidal behavior).

The present study demonstrates that number of somatic symptoms reported by adolescents with depression is closely related to the severity of adult psychiatric diagnoses in a stepwise manner. This finding shows that somatic symptoms in adolescent depression do not reflect transient problems. Mental health problems remain with increasing severity for each somatic symptom experienced during adolescence. The quarter of the adolescents that suffered from more than four somatic symptoms had a particularly poor outcome with high rates of severe mental disorders (e. g., suicidal behavior, recurrent and chronic depression, bipolar disorders, psychotic disorders, and panic disorder). The strong prediction of poor mental health indicted by somatic symptoms is of great importance in health care. Patients with somatic symptoms without medical explanation are often considered to be problematic and to have health anxiety. Currently, preferred clinical management is aimed at minimizing the use of health care and avoiding iatrogenic illness [28]. This study cannot deny that health anxiety can play an important role in somatic symptoms. However, considering the poor prognosis for mental health, the need for patient health care should not be underestimated*.*

In the healthy adolescents with no lifetime experience of depression by age 16, somatic symptoms predicted depression and other mental disorders in adulthood. Somatic symptoms thus preceded depression in this group, consistent with results in some previous population-based prospective studies [14,29]. From baseline, rarely did depressed adolescents without somatic symptoms develop somatoform disorders (5.7%), and not significantly more often than controls without somatic symptoms (4.2%). These results do not indicate a bidirectional relationship of somatic symptoms and depression.

The finding that a few somatic symptoms in healthy adolescents predicted mental health disorders in adulthood indicates that somatic symptoms either reflect vulnerability for mood disorders or constitute a subclinical mood disorder, rather than being caused by depression.

When somatic symptoms were compared with depressive symptoms (DSM-IV criteria) in a regression analysis, depressive symptoms did not better predict depression and anxiety. In fact, abdominal pain and perspiration without exertion better predicted adult depression than all the investigated depression criteria. This is a surprising finding given that depression in adolescence strongly predicts depression in adulthood [23,30]. The fact that depression criteria did not better predict depression and anxiety than concurrent somatic symptoms in depressed adolescents suggests that depression and concurrent somatic symptoms share a common pathway for mental disorders. This also indicates that cognitive and affective depression criteria and somatic symptoms may be different expressions of a common disorder.

In further regression analysis, family adversities, adolescent behavioral problems, stressful relationships, and long-term depression were added. Long-term adolescent depression is thought to have a toxic effect on the brain and in previous studies has been strongly associated with poor adult mental health [23,32]. In this analysis, abdominal pain in adolescence was a strong predictor of adult depression and anxiety and a predictor equally as good as an adolescent depressive episode most of the time of at least one year*.* Another population study also found that children with abdominal pain are at increased risk for adult mental disorders [31]. The strong predictive power of abdominal pain shows that not only number but also certain individual somatic symptoms are important. The unexpectedly strong influence of abdominal pain is not easily explained, and further research is needed. The predictive link between abdominal pain, perspiration without exertion, and future mood disorders poses a question for discussion. Which criteria would preferably be included in the diagnosis of depression in the next diagnostic manual?

Suicidal behavior in adulthood was common in adolescents with multiple (>5) somatic symptoms. This supports some earlier studies that found a relationship between somatic symptoms and suicidal thoughts [33,34]. The finding that somatic symptoms in adolescence predict future suicidal attempts even several years in the future has, to our knowledge, not been described previously. The group of depressed adolescents without somatic symptoms, which had a better prognosis for mental disorders, still had more suicidal behavior as well as alcohol abuse. This subgroup might be unique and represent a severe risk of suicidal behavior in a way we cannot explain.

Different studies taken together indicate a possible link between somatic symptoms, depression, and suicidal behavior. This link could be attributed to low-grade inflammation. The inflammation in depression, which is characterized by increased levels of cytokines like interleukin-1 and 6 (IL-1*,* IL-6), and tumor necrosis factor (TNF), may cause the occurrence of somatic symptoms of depression [35,36]. The recent finding of pathologically high levels of cytokines in the brain (IL-6) of suicide attempters links somatic symptoms with suicidal behavior and severity of depression [37]. Pro-inflammatory cytokines like IL-6 enhance the catabolism of L-tryptophan, which lowers the levels of serotonin in the central nervous system [38]. It is known that low levels of L-tryptophan, a precursor of serotonin in cerebrospinal fluid (CSF), can predict future suicide in suicide attempters [39]. One of several possible hypotheses is that the number of somatic symptoms in depression reflects increasing levels of IL-6 in the brain, which correlates with suicidal behavior caused by low levels of intracerebral serotonin.

Patients with somatic symptoms are typically categorized into different somatoform disorders. These disorders have been the subject of criticism by both professionals and patients. The diagnoses can cause confusion and offer little information about treatment or clinical guidance [28]. To offer new opportunities for research and treatment, it has been suggested that somatoform disorders be abandoned and instead somatic symptoms be included in DSM-V axis III as functional somatic symptoms [28]. Results from our study are in line with this. The present study and our previous cross-sectional study suggest that a depression diagnosis would benefit from including number of somatic symptoms as a marker of the severity of current depression [14] and as a prognostic marker of future mental disorders and suicidal behavior.

The clinical implication of this study is that adequate treatment guidelines are needed for patients with somatic symptoms. The prognosis for mental disorders is as poor for adolescents with depression and several somatic symptoms as for those with long-term adolescent depression. Even in healthy adolescents without a lifetime history of depression, somatic symptoms predict future mental disorder.

## Limitations

In the baseline study, somatic symptoms were assessed using a questionnaire and not followed by an interview or medical examination. Although physical disease is not as common in adolescents as in adults, some of the symptoms might have been explained by medically defined disorders. Hence, the validity could have been affected.

In our previous study of adolescents, we used a case-control design with subjects matched by age, sex, and school class [14]. Despite less power in the calculations, the design enabled us to more clearly investigate the differences between groups. Because of attrition at follow up, the case-control design was abandoned, and the calculations were performed on all adolescents followed up as adults. Thus, the differences between controls and subjects with adolescent depression could have been underestimated.

Investigation of a causal relationship was performed with different assessments in adolescence and adulthood. In adolescence, somatic symptoms were self-rated. In the follow-up, somatic symptoms were diagnosed through a clinical interview with the MINI, a screening instrument that identifies somatoform disorders. Because somatoform disorders have high thresholds, we cannot completely rule out that depressed adolescents without somatic symptoms more often suffered from individual somatic symptoms in the follow-up compared to the controls.

## Conclusions

Somatic symptoms in adolescence can predict severe mental health disorders in adulthood. Several somatic symptoms concurrent with adolescent depression are strongly linked to later high rates of suicidal attempts, bipolar disorders, psychotic disorders, post-traumatic stress disorder, recurrent depression, and chronic depression. Thus, effective treatment guidelines are needed for patients with somatic symptoms.

## Competing interests

The authors declare that they have no competing interests.

## Authors' contributions

HB conceived the original idea for this study, had primary responsibility for the data analysis, and wrote the manuscript. UJ together with HB had primary responsibility for enrollment and outcome assessment in the follow-up study, and contributed to the analyses and interpretation of the data and to the revision of the manuscript. GO had primary responsibility for protocol development, patient screening, and enrollment in the baseline study, and contributed to the revision of the manuscript. AP contributed to the analyses and interpretation of the data and to the revision of the manuscript. LvK contributed to the analyses and interpretation of the data and to the revision of the manuscript. ALvK supervised the design and execution of the study, and contributed to the revision of the manuscript. All authors approved the final version of the manuscript.

## Pre-publication history

The pre-publication history for this paper can be accessed here:

http://www.biomedcentral.com/1471-244X/12/90/prepub
